# Chromosome-level genome assembly of the western flower thrips *Frankliniella occidentalis*

**DOI:** 10.1038/s41597-024-03438-2

**Published:** 2024-06-04

**Authors:** Wei Song, Li-Jun Cao, Jin-Cui Chen, Wen-Xue Bao, Shu-Jun Wei

**Affiliations:** 1 Institute of Plant Protection, Beijing Academy of Agriculture and Forestry Sciences, Beijing, 100097 China; 2 College of Forestry, Inner Mongolia Agricultural University, Hohhot, 010019 China

**Keywords:** Entomology, Genomics

## Abstract

The western flower thrips *Frankliniella occidentalis* (Thysanoptera: Thripidae) is a global invasive species that causes increasing damage by direct feeding on crops and transmission of plant viruses. Here, we assemble a previously published scaffold-level genome into a chromosomal level using Hi-C sequencing technology. The assembled genome has a size of 302.58 Mb, with a contig N50 of 1533 bp, scaffold N50 of 19.071 Mb, and BUSCO completeness of 97.8%. All contigs are anchored on 15 chromosomes. A total of 16,312 protein-coding genes are annotated in the genome with a BUSCO completeness of 95.2%. The genome contains 492 non-coding RNA, and 0.41% of interspersed repeats. In conclusion, this high-quality genome provides a convenient and high-quality resource for understanding the ecology, genetics, and evolution of thrips.

## Background & Summary

Thrips are a group of tiny insects from the order Thysanoptera. Most thrips species feed on plants and fungi, while only a small number of species are predators of small invertebrates^1^. There are over 7000 species of thrips, with 150 of them being harmful to plants^2^. Pest thrips are causing increasing damage to crop production worldwide^3,4^. Thrips can be easily dispersed through transportation of host plants^5^. The western flower thrips (WFT) *Frankliniella occidentalis* is one of the most notorious thrips worldwide^6,7^. This species is native to America and has dispersed worldwide since the 1970s as an invasive species^8^. The invasion genetics of this species have been widely investigated^9–12^. Insecticides were frequently used to control this pest and thus causing pesticide resistance in the field^13,14^. However, resistant mechanisms of WFT to many insecticides remain to be explored^14,15^. In addition to directly feeding on plants, WFT can transmit plant viruses from the genus *Tospovirus*, making it an important species to understand insect-plant-virus interaction^16,17^. A genome assembly is crucial to understand the complex biology, ecology and genetic of the WFT. The WFT genome is the first that has been assembled in thrips and made publicly available^18^, providing invaluable resources for studying the genetic mechanisms governing pest and vector biology, feeding behaviours, ecology, and resistance to insecticides and development of novel control methods^10,19,20^. However, some genes are scattered across different scaffolds of the currently assembled genome, which hinders the functional genomics study of this species. An improved genome assembly of WFT to a chromosome level will benefit future studies of this important insect pest. Here, we assembled the previously published scaffold-level genome of WFT to a chromosomal level using chromosome conformation capture (Hi-C) technology^18^.

## Methods

### Sample collection and Hi-C library sequencing

The chromosome conformation of the genome was analysed to determine the order and orientation of the contigs using Hi-C technology. A strain of WFT was reared for approximately 10 generations and used for Hi-C library construction at the College of Forestry, Inner Mongolia Agricultural University, Hohhot, China. Approximately 1000 live adults of mixed sex were ground and then cross-linked in a fresh, ice-cold nuclear isolation buffer with a 2% formaldehyde solution for 10 minutes. The fixed cells were then digested using *DpnII* (NEB) enzymes, and further processed by cell lysis, incubation, DNA end labelling with biotin-14-dCTP, and blunt-end ligation of crosslinked fragments. The Hi-C library was amplified by 12–14 PCR cycles and sequenced on the Illumina NovaSeq 6000 platform. A total of 36.11 Gb of clean data were generated, representing 119.34X coverage of the genome.

### Genome characteristics estimation

**Fig. 1:**
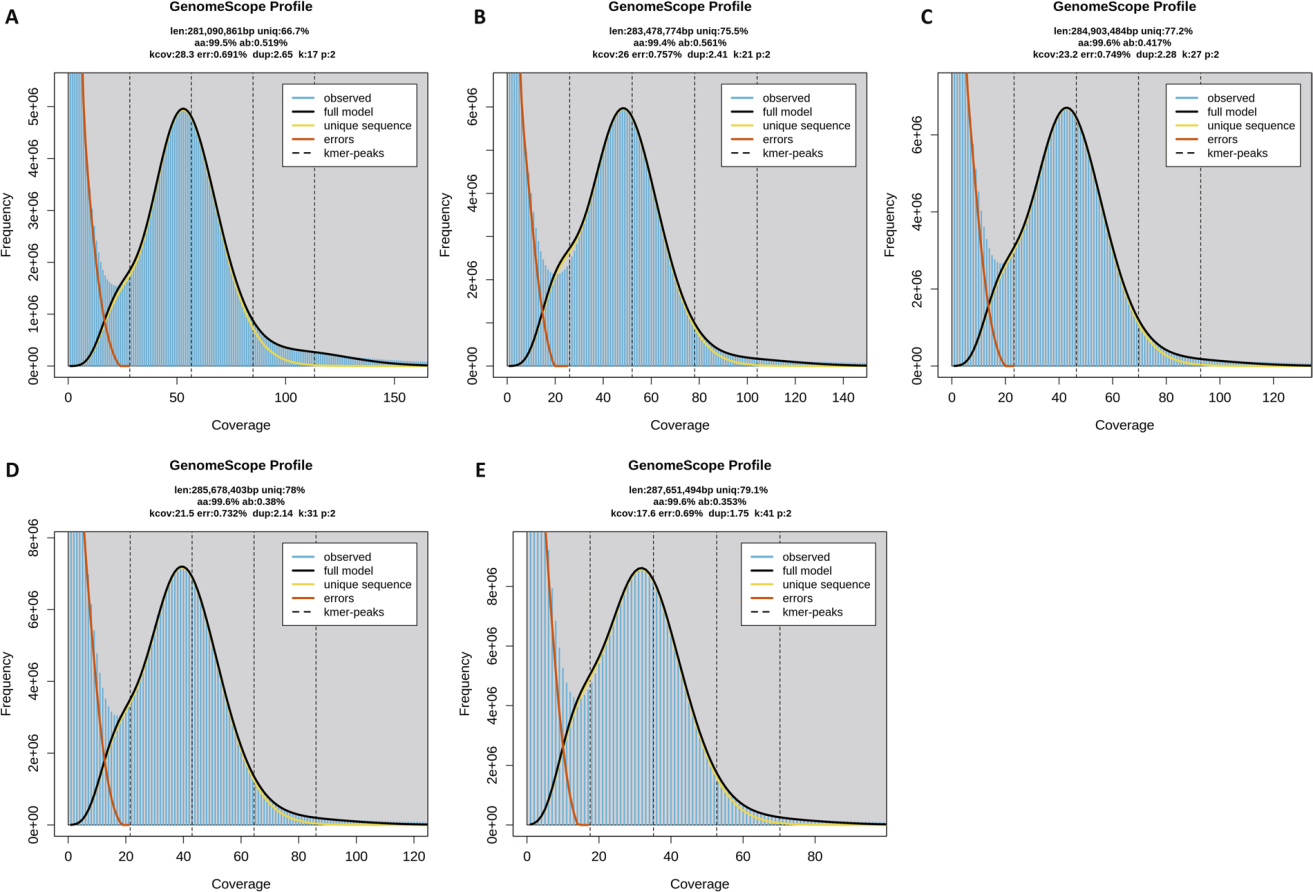
Estimated characteristics of *Frankliniella occidentalis* genome based on Illumina short-read data. Results were obtained in KMC v3.0 and GCE v2.0 with 17- (**A**), 21- (**B**), 27- (**C**), 31- (**D**) and 41- (**E**) mer. len, estimated genome size in bp; aa, homozygosity rate; ab, heterozygosity rate; dup, duplication rate.

Genome characteristics were estimated based on Illumina short-reads. Raw reads of the whole genome sequencing of WFT were downloaded from the NCBI Sequence Read Archive database (accession number of SRR1300140). The raw sequences were trimmed using the software fastp^21^ with default parameters. The trimmed data was used to count the *K*-mer distribution histogram under 17, 21, 27, 31 and 41-mer using KMC v3.0^22^ with parameters ‘-m96 -ci1 -cs10000’ and ‘-cx10000’. The genome size, heterozygosity rate, and duplication rate were estimated using GCE v2.0^23^ with default parameters. The estimated genome size and genome duplication decreased as the *K*-mer increased, ranging from 281 Mb to 287 Mb and 1.75% to 2.65%, respectively. Each *K*-mer distribution showed single-peak, indicating that the genome of WFT is a simple one (Table 1, Fig. 1).

**Table 1:** Statistics for chromosomal-level assembly and annotation of *Frankliniella occidentalis* genome.

Type	Item	Feature
Genome survey	Genome size (Mb)	281.09–287.65
	Error rate	0.690%–0.757%
	Duplicated sequence	1.75%–2.65%
Genome feature	Genome size (Mb)	302.58
	Chromosome number	15
	Contig number	250191
	Longest scaffold (Mb)	32.461
	Shortest scaffold (Mb)	15.116
	Rate of N	37.16%
	GC content	50.75%
	Scaffold N50 (Mb)	19.071
	Contig N50 (bp)	1533
	BUSCO completeness	C:97.8% [S:96.8%, D:1.0%], F:1.1%, M:1.1%, n:1367
Protein-coding gene	Gene number	16312
	BUSCO completeness	C:95.2% [S:93.7%, D:1.5%], F:1.6%, M:3.2%, n:1367

### Genome assembly and annotation

The scaffold-level genome was downloaded from NCBI database (accession number: GCF_000697945) and used for chromosomal-level genome assembly based on Hi-C sequencing data. Low-quality reads and adapters from the Hi-C library were filtered using Trimmomatic v0.39^24^ with default parameters and then mapped to the genome contigs using Juicer^25^ with default parameters. The reads were grouped into chromosomes using 3D *de novo* assembly (3D-DNA)^26^ with parameters ‘–editor_repeat_coverage = 15, -r 2’. Error joints were manually adjusted in Juicebox v2.16.00 (https://github.com/aidenlab/Juicebox), and the raw-chromosomes were updated using the script “run-asm-pipeline-post-review.sh” in 3D-DNA again.

**Fig. 2:**
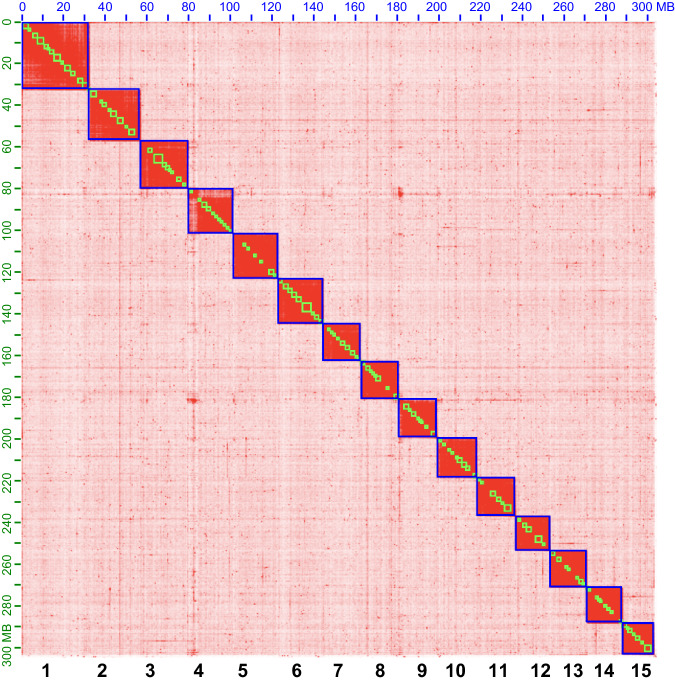
Genome-wide contact matrix of *Frankliniella occidentalis* generated using Hi-C data. Each blue square represents a chromosome, each green square represents a contig. Fifteen chromosomes were anchored under the default parameters of Juicer and 3D-DNA software. Numbers on the axes show the chromosome length in Mb. The numbers in bold at the bottom of the figure represents the chromosomes number.

The repeat-masked genome assembly was submitted to the online tool Helixer^27^ for genome structure annotation under the invertebrate lineage-specific mode. Helier is a novel tool for cross-species gene annotation of large eukaryotic genomes using deep learning algorithms. Functional annotation was performed by BLAST the proteins against the EggNOG v5.0^28^ database using eggNOG-Mapper^29^. Additionally, the entire gene sets were functionally annotated by aligning protein sequences with the Nr database, Uniport_SwissProt, Uniref90, InterPro (-appl pfam, PRINTS, PANTHER, ProSiteProfiles, SMART, CDD, SFLD, AntiFam), KEGG and GO database using BLASTP and InterProScan version 5.59–91.0 (https://github.com/ebi-pf-team/interproscan) with an e-value cutoff of e < 10^−5^. The final genome assembly was consisted of 250,191 contigs, which were assembled into 15 chromosomes (Fig. 2). The chromosome sizes ranged from 15.116 Mb to 32.461 Mb, with a total length of 302.58 Mb, a contig N50 length of 1533 bp, and a scaffold N50 length of 19.071 Mb. We numbered the chromosomes in descending order of their size. Compared to the scaffold-level assembly with a size of 415.8 Mb and scaffold N50 of 948.9 kb, the genome size was reduced and became more approximated to the estimated genome size. In total, 16,312 protein-coding genes (PCGs) were identified, which is 547 genes fewer than the official gene set (OGS v1.0) of 16,859 for the scaffold-level assembly. The functionally annotated terms were discrepant according to the reference databases, ranged from 15619 PCGs for Nr database to 370 domains for InterPro database (Table 2). The G + C content of the final genome assembly was 50.75% (Table 1), which is similar to that of the published WFT genome^18^, lower than that of *Frankliniella intonsa*^30,31^, *Megalurothrips usitatus*^32,33^, *Stenchaetothrips biformis*^34^ and *Thrips palmi*^35^, while slightly higher than those of *Frankliniella fusca*^36^.

**Table 2:** Summary of annotated protein-coding genes in *Frankliniella occidentalis* genome.

Database	Number	Percentage %
Nr terms	15619	95.75
Uniref90 terms	15413	94.48
EggNOG	9994	61.26
Uniprot/Swissprot terms	9230	56.58
KEGG terms	9027	55.34
GO terms	2301	14.10
InterPro domains	370	2.27

Percentage, percentage of each item in all genes.

### Repeat elements and non-coding RNA predictions

The repetitive elements longer than 1000 bp were identified against the Insecta repeats within RepBase Update (20120418). The identification was performed using RepeatMasker version open-4.0.0^37^ (-no_is -norna -xsmall -q) under the search engine RM-BLAST (v2.2.23+). *De novo* identification of transposable elements (TEs) was performed using RepeatModeler^38^. Non-coding RNAs were identified using Rfam^39,40^, ribosome RNAs (rRNAs) and transfer RNAs (tRNAs) were searched by tRNAscan-SE v2.0^41^ and RNAmmer v1.2^42^, both with default parameters. A total of 189159 transposable elements (TEs) were identified, including 5005 retroelements with a total length of 542068 bp, 8850 DNA transposons and 175304 Tandem Repeats (TRs) (Table 3). We identified 49 miRNAs, 19 snRNAs, 31 snoRNAs, 101 rRNAs and 292 tRNAs in WFT genome (Table 4).

**Table 3:** Repeated elements identified in the *Frankliniella occidentalis* genome.

Element	Number of elements*	Length of occupied (bp)	Percentage sequence (%)
**Retroelements**	5005	542068	0.18
SINEs:	81	5370	0
Penelope	657	37141	0.01
LINEs:	2719	262776	0.09
L2/CR1/Rex	366	71210	0.02
R1/LOA/Jockey	738	62368	0.02
R2/R4/NeSL	29	2911	0
RTE/Bov-B	31	3604	0
L1/CIN4	15	1031	0
LTR elements:	2205	273922	0.09
BEL/Pao	204	14457	0
Ty1/Copia	566	94436	0.03
Gypsy/DIRS1	1404	162337	0.05
**DNA transposons**	8850	609921	0.2
hobo-Activator	1017	70018	0.02
Tc1-IS630-Pogo	3838	234099	0.08
PiggyBac	88	5746	0
Tourist/Harbinger	26	2194	0
Other (Mirage, P-element, Transib)	27	2489	0
**Rolling-circles**	767	47938	0.02
**Unclassified:**	987	86204	0.03
**Total interspersed repeats:**		1238193	0.41
**Small RNA:**	118	8152	0
**Satellites:**	22	3419	0
**Simple repeats:**	175282	7582661	2.51
**Low complexity:**	29311	1534395	0.51

## Data Records

The genome project was deposited at NCBI under BioProject No. PRJNA1016120. The Hi-C sequencing data were deposited in the Sequence Read Archive at NCBI under accession SRR26106059^43^. The genome assembly, genome structure annotation and protein files were deposited in Figshare under a DOI of 10.6084/m9.figshare.24968679.v1^44^. The final genome assembly was also deposited in GenBank at NCBI under the accession number GCA_035583395.1^45^.

## Technical Validation

Benchmarking Universal Single-Copy Orthologs (BUSCO) v5.4.5^46^ was used to estimate the integrity and quality of the genome assembly and the annotated protein-coding genes based on the Eukaryota, Metazoa, Arthropoda and Insecta (odb_10, released on 2024-01-08) datasets. For the chromosome-level genome assembly, the BUSCO completeness was 97.7%, 98.7%, 98.5% and 97.8% based on the Eukaryota, Metazoa, Arthropoda and Insecta datasets, respectively. For the protein-coding gene set, the BUSCO completeness was 93.3%, 95.6%, 95.7% and 95.2% based on the Eukaryota, Metazoa, Arthropoda and Insecta datasets, respectively. To avoid the genetic differences of samples for assembly, we mapped the Illumina short-reads for scaffold-level assembly and Hi-C library sequencing reads obtained in our study to our assembled chromosome-level genomes using BWA version 0.7.17-r1198-dirty^47^. The mapping rate of Illumina short-reads and Hi-C sequencing data was 94.70% and 95.15%, respectively.

**Table 4:** Non-coding RNA identified in the *Frankliniella occidentalis* genome.

Class		Number
miRNA Count		49
snRNA Count		19
snoRNA Count		31
rRNA count	5s_rRNA	63
	8s_rRNA	38
tRNA count	tRNAs decoding Standard 20 AA	142
	Selenocysteine tRNAs	1
	tRNAs with undetermined/ unknown isotypes	9
	Predicted pseudogenes	140
	Total tRNAs	292
	tRNAs with intron	12

## Data Availability

No specific code or script were used in this study.
